# Cost-Effectiveness of Nationwide HPV Vaccination in Girls in Kazakhstan: A UNIVAC-Based Analysis

**DOI:** 10.3390/vaccines14050453

**Published:** 2026-05-19

**Authors:** Raikhan Nissanova, Markhabat Kassenov, Vladislava Suchshikh, Perizat Akshalova, Zhandos Abay, Vladimir Kirpichenko, Aiken Karabassova, Saira Kaimoldina, Zhibek Zhetpisbay, Elvira Bashenova, Ainur Nurpeisova

**Affiliations:** 1 Kazakh Scientific Research Veterinary Institute, 223 Rayymbek Avenue, Almaty 050016, Kazakhstan; kasenovmarhabat@gmail.com (M.K.); vladasali@gmail.com (V.S.); peri.akshalova@gmail.com (P.A.); abai.zhandos15@gmail.com (Z.A.); vladimir.kirpichenko1992@gmail.com (V.K.); aiken.karabasova@gmail.com (A.K.); kaimoldina84mir@gmail.com (S.K.); elvirabashenova17@gmail.com (E.B.); 2 Department of Computer Science, Faculty of Information Technology, Al-Farabi Kazakh National University, Almaty 050040, Kazakhstan; zhibekzhetpisbay@gmail.com

**Keywords:** HPV vaccination, cost-effectiveness analysis, cervical cancer prevention, UNIVAC, DALYs, ICER, Gardasil-4, Kazakhstan

## Abstract

**Background**: Cervical cancer, largely attributable to persistent infection with high-risk human papillomavirus (HPV), remains a major public health burden worldwide, including in Kazakhstan, where limited screening coverage and low public awareness contribute to substantial incidence and mortality. This study evaluated the cost-effectiveness and epidemiological impact of a nationwide HPV vaccination programme for 10-year-old girls in Kazakhstan using the quadrivalent Gardasil-4 vaccine. **Methods**: A 10-year modelling analysis (2025–2035) was conducted using the World Health Organization (WHO)-endorsed Universal Vaccination Impact and Cost-Effectiveness Assessment (UNIVAC) tool adapted to Kazakhstan-specific epidemiological and economic parameters. Vaccination coverage was projected at 98.0% for the first dose and 96.5% for the second dose. Incremental cost-effectiveness ratios (ICERs) and disability-adjusted life years (DALYs) averted were estimated from governmental and societal perspectives. Sensitivity analyses assessed uncertainty in vaccine coverage, vaccine costs, and epidemiological inputs. **Results**: Over the 10-year period, the vaccination programme was projected to reduce HPV-related disease cases by 68.2% (from 112,198 to 35,628) and deaths by 68.3% (from 15,921 to 5056), while averting 67,445 DALYs. The ICER was estimated at US$ 533 per DALY averted from the governmental perspective and US$ 1169 from the societal perspective. Projected healthcare cost savings reached US$ 42.8 million, driven largely by reductions in 21,748 hospitalisations and 13,706 outpatient visits. These findings remained robust in probabilistic sensitivity analysis, with the probability of cost-effectiveness increasing as the willingness-to-pay threshold rose. **Conclusions**: UNIVAC-based modelling suggests that introduction of a national HPV vaccination programme for 10-year-old girls in Kazakhstan using Gardasil-4 could substantially reduce cervical cancer burden and related mortality while generating considerable healthcare savings. These findings support the cost-effectiveness of nationwide HPV vaccination in Kazakhstan.

## 1. Introduction

Human papillomavirus (HPV) infection remains a major cause of cervical cancer and related mortality among women worldwide [1,2,3]. According to the World Health Organization (WHO), more than 350,000 women have died from cervical cancer, the vast majority of cases being attributable to persistent infection with oncogenic HPV types [4,5,6]. Despite advances in screening and vaccination [7,8], the burden of cervical cancer remains substantial across regions, with marked geographic variation in incidence, mortality, and genotype distribution [9,10,11]. These disparities are particularly pronounced in low- and middle-income countries, where limited access to preventive services and delayed implementation of vaccination programmes continue to drive disease burden [12].

In response to this global challenge, the WHO has launched the cervical cancer elimination strategy, targeting 90% HPV vaccination coverage, 70% screening coverage, and 90% access to treatment by 2030. Achieving these targets requires not only effective implementation of vaccination programmes but also robust country-specific evidence to support policy decisions, particularly in settings with constrained resources and heterogeneous epidemiological profiles [13,14].

HPV infection remains highly prevalent in Kazakhstan, with reported prevalence estimates ranging from 43.8% to 62.4% among women attending gynecological clinics and those with abnormal cervical cytology, based on studies conducted between 2018 and 2022 [15,16,17]. Cervical cancer is a major public health burden globally, with over half a million new cases and 300,000 deaths annually, primarily affecting low-income and middle-income countries lacking screening and vaccination programmes [18]. Two cervical cancer screening approaches are currently in use in Kazakhstan: a nationwide screening programme for women aged 30–70 years and an opportunistic screening pathway for older women attending gynecological consultations [19,20,21]. Pap smear screening using the Bethesda system has been implemented since 2011, and liquid-based cytology was introduced in 2014; however, screening coverage remains limited, reaching only 45.9% of the target population [22,23]. These data indicate a substantial residual burden of HPV-associated disease despite the existing preventive framework [24,25], and highlight the need for complementary primary prevention strategies.

HPV vaccination is a key primary prevention strategy for reducing HPV infection and the incidence of HPV-associated cervical lesions and cervical cancer [26,27,28]. The quadrivalent Gardasil-4 vaccine provides protection against HPV types 6, 11, 16, and 18 and has demonstrated high efficacy in randomized controlled trials and post-licensure studies conducted among adolescent girls and young women prior to sexual debut in studies published between 2007 and 2022 [29,30,31]. The population-level impact of vaccination, however, depends strongly on vaccine uptake, programme delivery [32], and long-term coverage, with broader benefits expected where vaccination is implemented before sexual debut and sustained at high coverage levels, typically exceeding 80–90% of the target population, as commonly assumed in population-level HPV vaccination models [33,34,35]. In addition, integration of vaccination with screening strategies may further reduce the lifetime risk of cervical cancer [36,37,38]. Importantly, in countries with suboptimal screening coverage, vaccination may play a disproportionately important role in reducing long-term disease burden [39].

In Kazakhstan, the implementation of HPV vaccination has been particularly challenging. A pilot vaccination programme was suspended in 2017 because of widespread parental refusal, primarily associated with concerns about vaccine safety, insufficient awareness, and misinformation, highlighting the importance of public confidence and effective health communication [38]. In 2024, HPV vaccination in girls with the quadrivalent Gardasil-4 vaccine was reintroduced, based on global WHO recommendations and available epidemiological evidence on HPV burden, creating a renewed need for country-specific data to inform policy and programme sustainability. The selection of the quadrivalent vaccine reflects its current use in the national immunization programme [28,40]. In such contexts, where real-world data are limited and programme performance is uncertain, modelling approaches provide a critical tool for estimating the potential epidemiological and economic impact of vaccination under defined scenarios.

In settings where local epidemiological and economic data are limited, decisions on vaccine introduction and scale-up may be hindered by uncertainty regarding expected health benefits, affordability, and long-term value [41,42,43]. Policy-oriented modelling tools, such as the WHO-endorsed UNIVAC framework, are specifically designed to support evidence-informed decision-making by integrating demographic, epidemiological, and economic data to simulate vaccination scenarios and estimate their potential impact at the population level.

Therefore, this study aimed to evaluate the epidemiological impact and cost-effectiveness of nationwide HPV vaccination in 10-year-old girls in Kazakhstan using the WHO-endorsed UNIVAC model. Specifically, we estimated the potential reductions in HPV-related disease burden, deaths, and healthcare utilization, as well as the incremental cost-effectiveness of the programme from governmental and societal perspectives. This analysis is intended to provide scenario-based evidence to inform national immunization policy and support the sustainable implementation of HPV vaccination in Kazakhstan.

## 2. Materials and Methods

The Universal Vaccination Impact and Cost-Effectiveness Assessment (UNIVAC) tool, developed by the WHO, was used to assess the cost-effectiveness of HPV vaccination in girls in Kazakhstan. The model supports national-level decision-making regarding the implementation of vaccination programmes by estimating the direct effects of vaccination on morbidity and mortality and by calculating the incremental cost-effectiveness ratio (ICER) using epidemiological and economic parameters.

### 2.1. Experimental Design

A static cohort-based modelling analysis using the WHO-endorsed UNIVAC framework approach using the UNIVAC tool (version 1.4.63, WHO, Geneva, Switzerland) [44] was employed to evaluate the cost-effectiveness of the HPV vaccination programme in Kazakhstan. The model simulated cohorts of girls starting at age 10 years, covering annual birth cohorts from 2015 to 2024, with a 10-year analytic period (2025–2035). The programme involved a two-dose immunization regimen using the Gardasil-4 vaccine (Merck & Co., Rahway, NJ, USA), with the first dose administered at age 10 years and the second dose given 6 months later (at 10.5 years). This approach allowed the evaluation of both the direct effects and the longer-term impact of vaccination over the 10-year analytic horizon, capturing the gradual accumulation of health and economic benefits in vaccinated cohorts under high coverage conditions (98.0% for the first dose and 96.5% for the second dose). While vaccination cohorts were modelled during the 2025–2035 implementation period, the reported reductions in cervical cancer cases, deaths, and DALYs represent lifetime projected outcomes for the vaccinated cohorts generated within the UNIVAC framework.

### 2.2. Data Adaptation and Scope of the Study

Baseline data were adapted for Kazakhstan, which the World Bank classifies as an upper-middle-income country [45]. The analysis used WHO European Region (EUR) parameters with demographic adjustments based on average infant mortality rates reported by the United Nations Population Division (UNPOP) [46]. The modelling period covered 10 years, from 2025 to 2035, with vaccination of cohorts born between 2015 and 2024 modelled, corresponding to vaccination at age 10 years during the period 2025–2034. The minimum vaccination age was set at 10 years.

### 2.3. Vaccination Protocol

The quadrivalent Gardasil-4 vaccine targeting four HPV serotypes was selected for this study. A two-dose immunization regimen was adopted in accordance with WHO recommendations [47], with the first dose administered at age 10 years and the second dose at age 10.5 years. Routine vaccination of the target population was assumed, delivered through the existing school-based immunization platform alongside other routine childhood and adolescent vaccines. The quadrivalent Gardasil-4 vaccine was selected based on its current use in Kazakhstan following the reintroduction of the national HPV vaccination programme in 2024, ensuring alignment of the model with the existing immunization strategy and enhancing the policy relevance of the analysis. This approach is consistent with WHO recommendations for country-specific immunization modelling.

### 2.4. Disease Categories and Epidemiological Data

The analysis covered HPV-related outcomes including low-grade cervical intraepithelial neoplasia (CIN1), high-grade cervical intraepithelial neoplasia (CIN2/3), localized and regional cervical cancer, and cervical cancer mortality. Cervical cancer incidence and mortality data were obtained from the GLOBOCAN 2020 database [48]. Estimates for CIN1 and CIN2/3 were derived from published literature and adapted to the Kazakhstan population structure within the UNIVAC modelling framework. These estimates were aligned with the country’s age structure and epidemiological profile to improve modelling accuracy. CIN1 incidence peaked in the 25–30-year age group at 92.8 cases per 100,000 population. The incidence of CIN2/3 was highest in the same age group at 31 cases per 100,000 population and decreased with age. The incidence of cervical cancer peaked at 19.8 and 15.7 cases per 100,000 population for localized and regional stages, respectively, among women aged 45–50 years.

### 2.5. Economic Evaluation

Cost-effectiveness was assessed from governmental and societal perspectives. The analysis included costs related to vaccine procurement, logistics, storage, personnel, and treatment of HPV-related diseases. Future costs and benefits were discounted at an annual rate of 3%, with 2025 used as the base year for calculations. The vaccine price was set at US$ 12.20 per dose, reflecting current market conditions and contracted prices in countries with a comparable income level. Additional costs for consumables (syringes and disposal containers) and logistics were estimated using current market data. The 3% discount rate was applied in accordance with international recommendations for economic evaluations. From the governmental perspective, costs were assumed to be covered by the public healthcare system, while the societal perspective reflects a broader analytical framework without explicit inclusion of indirect costs. Accordingly, healthcare cost savings were conservatively reported only for the governmental perspective, while societal perspective estimates were limited to incremental cost-effectiveness comparisons without monetized indirect benefits.

### 2.6. Key Assumptions

Only direct effects of vaccination, including reductions in morbidity and mortality, were considered. Indirect effects, such as herd immunity and type replacement, were excluded in order to provide conservative estimates.

### 2.7. Vaccination Coverage

Projected coverage rates were 98.0% for the first dose and 96.5% for the second dose, based on WUENIC 2021 estimates for routine childhood immunization coverage in Kazakhstan, used as a proxy for a high-performance HPV vaccination scenario. Annual cohorts of vaccinated girls born between 2015 and 2024 were modelled, with vaccination continuing until 2034. The assumed coverage levels represent a high-performance implementation scenario and were based on WUENIC estimates and WHO modelling guidance. These assumptions were selected to reflect the potential impact of vaccination under optimal programme conditions. To address uncertainty and enhance robustness, vaccination coverage parameters were also varied within the probabilistic sensitivity analysis, including lower-coverage scenarios. Coverage rates were varied within plausible ranges (e.g., ±10–20%) in sensitivity analyses.

### 2.8. Cost Analysis

The vaccine cost for Gardasil-4 was US$ 12.20 per dose (range: US$ 4.50–25.00). Syringe cost was US$ 0.07 per unit, and disposal container cost was US$ 0.01 per unit. International logistics were estimated at 10% of vaccine cost. Vaccine and consumable wastage was assumed to be 5% (range: 4.0–6.0%).

### 2.9. Healthcare Expenditure

Estimated outpatient costs for CIN1/CIN2/3 ranged from US$ 50 to US$ 100 per visit. The outpatient cost associated with localized cervical cancer was estimated at US$ 6321 per visit. Hospitalization costs for CIN1/CIN2/3 were estimated at US$ 6321. These cost estimates were derived from publicly available data sources and published literature, supplemented by assumptions consistent with comparable healthcare settings.

### 2.10. Sensitivity Analysis

A probabilistic sensitivity analysis (PSA) was performed to account for parameter uncertainty. Variations in vaccination coverage, vaccine costs, and epidemiological parameters were incorporated to assess their impact on ICER estimates and the robustness of the model outcomes.

## 3. Results

This study evaluated the cost-effectiveness of HPV vaccination in Kazakhstan using the quadrivalent Gardasil-4 vaccine over the period 2025–2034. The results are presented from governmental and societal perspectives (Table 1).

According to the model, the cost per DALY averted was estimated at US$ 533 from the governmental perspective and US$ 1169 from the societal perspective. From the governmental perspective, the vaccination programme was associated with healthcare cost savings of US$ 42,856,372, whereas from the societal perspective no direct healthcare cost savings were included in the model. In both perspectives, the total number of DALYs averted was 67,445, indicating a substantial projected reduction in the burden of HPV-related disease.

The implementation of HPV vaccination in Kazakhstan was projected to result in marked reductions in healthcare utilization and expenditure (Table 2).

As shown in Table 2, total healthcare costs were projected to decline from US$ 62,797,375 to US$ 19,941,003, corresponding to savings of US$ 42,856,372. Vaccination was also associated with a reduction in outpatient visits from 388,660 to 125,872. In addition, hospitalization-related costs were projected to decline from US$ 62,408,716 to US$ 19,815,222. These findings indicate a substantial reduction in healthcare system burden following the introduction of HPV vaccination.

The burden of HPV-related disease was also projected to decrease substantially following vaccine introduction. Overall, the total number of cases was estimated to decline from 112,198 to 35,628, representing a 68.2% reduction. Low-grade cervical lesions (CIN1) were projected to decrease from 60,248 to 19,132 cases, and high-grade lesions (CIN2/3) from 20,083 to 6377 cases. Localized cervical cancer cases were estimated to decline from 15,948 to 5064, while deaths were projected to decrease from 15,921 to 5056.

From the governmental perspective, the reduction in healthcare costs was driven largely by fewer hospitalizations and outpatient visits, corresponding to 21,748 fewer hospitalizations and 13,706 fewer outpatient visits over the modelled period. These changes indicate that the vaccination programme could substantially reduce resource use within the healthcare system.

Birth cohort analysis indicated that routine vaccination starting in 2025 among 10-year-old girls would result in an annual fully vaccinated population of approximately 364,639–374,651 individuals. Over the full analytic period, the programme was projected to require approximately 3.9 million vaccine doses. These findings provide an estimate of the scale of implementation required for nationwide vaccination.

The total discounted cost of the vaccination programme was estimated at US$ 78.83 million over the study period, including vaccine procurement and consumables. The cost per birth cohort was projected to decline from US$ 9.05 million in 2025 to US$ 6.87 million in 2034, indicating improved cost efficiency over time as the programme expanded.

The vaccination programme was projected to reduce the overall disease burden from 98,827 to 31,382 DALYs, corresponding to a net reduction of 67,445 DALYs. The largest reduction in DALYs was observed among routinely vaccinated cohorts between 2025 and 2034, consistent with the expected long-term public health benefit of sustained immunization.

**Figure 1:**
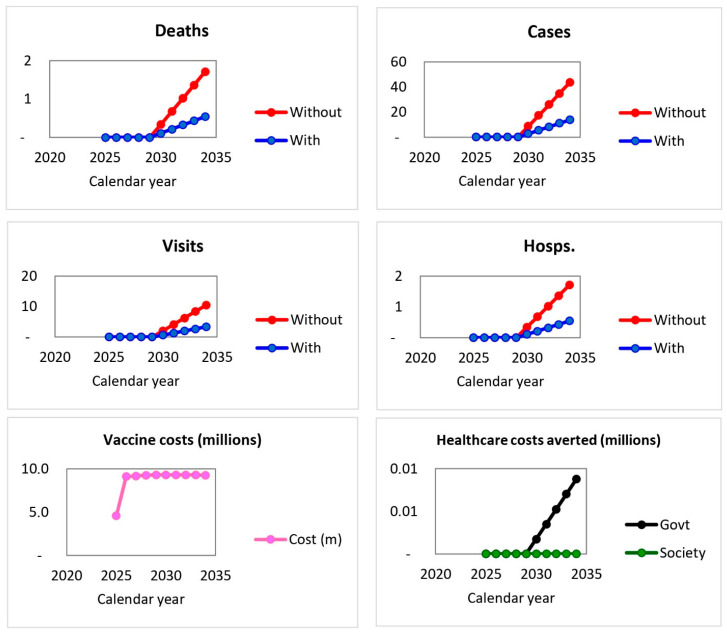
Trends in health and economic outcomes during the first decade of the HPV vaccination programme.

The temporal trends in health and economic outcomes are shown in Figure 1.

Figure 1 shows the annual dynamics of cases, deaths, outpatient visits, hospitalizations, vaccine programme costs, and healthcare costs averted under scenarios with and without vaccination. During the first years of programme implementation, differences between the two scenarios were minimal; however, from 2030 onward, increasing divergence became evident, with consistently lower numbers of cases, deaths, outpatient visits, and hospitalizations in the vaccination scenario. Vaccine programme costs remained relatively stable over time, whereas healthcare cost savings increased progressively as the health benefits of vaccination accumulated. Note: Hosps., hospitalizations.

The annual dynamics of these indicators are summarized in Table 3.

The data presented in Table 3 demonstrate the gradual accumulation of programme impact over time. No major differences between vaccination and no-vaccination scenarios were observed during 2025–2029. However, by 2030–2034, reductions in cases, healthcare utilization, and deaths became increasingly apparent in the vaccinated scenario. Healthcare cost savings also emerged during the later years of the model horizon, reflecting the delayed but cumulative benefits of vaccination.

**Figure 2:**
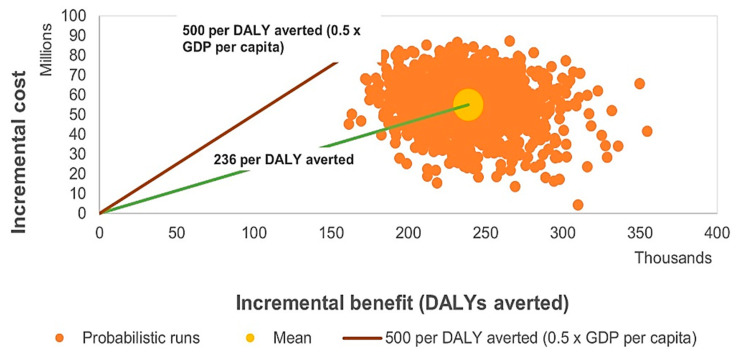
Incremental cost-effectiveness of Gardasil-4 vaccination in Kazakhstan from the governmental perspective.

**Figure 3:**
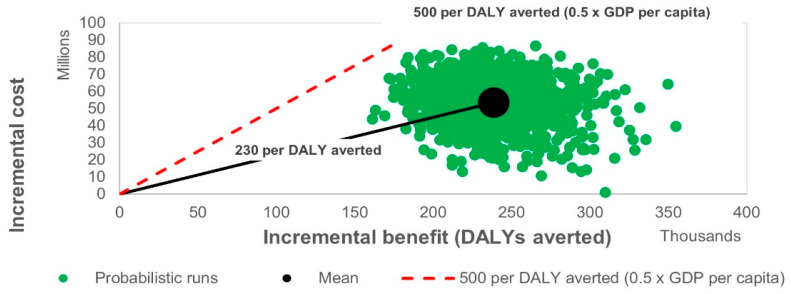
Incremental cost-effectiveness of Gardasil-4 vaccination in Kazakhstan from the societal perspective.

**Figure 4:**
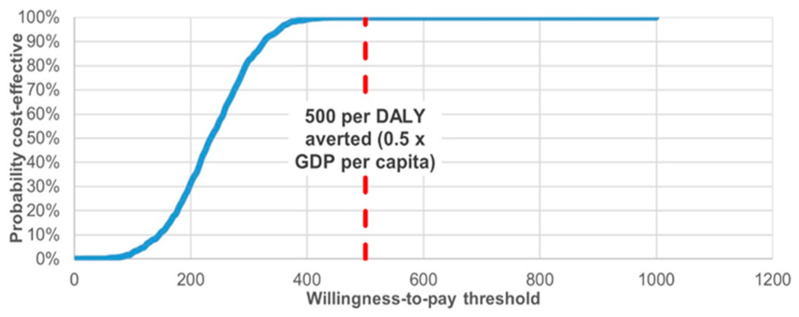
Probability that HPV vaccination is cost-effective at different willingness-to-pay thresholds from the governmental perspective.

To evaluate the robustness of the model under parameter uncertainty, a probabilistic sensitivity analysis (PSA) was performed. The results are presented in Figure 2, Figure 3 and Figure 4 as cost-effectiveness planes and cost-effectiveness acceptability curves. Figure 2 and Figure 3 illustrate modelled annual trends and mean projected values over the analytic horizon, whereas the ICER values reported in Table 1 represent the final base-case cost-effectiveness estimates derived from cumulative model outputs.

As shown in Figure 2, most probabilistic simulation points were located below the willingness-to-pay threshold of US$ 500 per DALY averted (0.5 × GDP per capita), indicating a high probability that HPV vaccination would be cost-effective from the governmental perspective.

Similarly, from the societal perspective, most probabilistic scenarios remained below the willingness-to-pay threshold of US$ 500 per DALY averted, supporting the economic robustness of the programme under varying assumptions.

From the governmental perspective, the cost-effectiveness acceptability curve indicated that the probability of HPV vaccination being cost-effective increased progressively with higher willingness-to-pay thresholds and approached 90% at approximately US$ 500 per DALY averted. The blue line represents the probability of cost-effectiveness, the red dashed line indicates the willingness-to-pay threshold.

Taken together, the PSA findings indicate that the projected cost-effectiveness of HPV vaccination in Kazakhstan remained robust across a range of plausible epidemiological and economic assumptions.

## 4. Discussion

The present study indicates that implementation of an HPV vaccination programme for girls in Kazakhstan using the quadrivalent Gardasil-4 vaccine would be associated with substantial epidemiological and economic benefits. The projected reductions in morbidity, mortality, and healthcare expenditure suggest that HPV vaccination could represent a cost-effective strategy for reducing the burden of HPV-associated disease in the country.

The projected decline in overall morbidity and mortality is broadly consistent with findings from international studies evaluating early HPV vaccination in adolescent girls [43,49]. In the present model, vaccination was associated with a 68.2% reduction in HPV-related disease and a 68.3% reduction in deaths over the analytic period. These estimates are in line with evidence from large-cohort studies in Europe and North America showing that early vaccination, particularly before the age of 14 years, is associated with marked reductions in cervical cancer risk and related precancerous lesions [50]. Reductions in CIN1 and CIN2/3 are particularly relevant, as prevention of precancerous lesions may decrease the need for invasive diagnostic and therapeutic procedures and reduce the long-term burden on both patients and healthcare systems [51].

The economic findings further support the potential public health value of the programme. The estimated cost per DALY averted was US$ 533 from the governmental perspective and US$ 1169 from the societal perspective, indicating favourable cost-effectiveness within the context of an upper-middle-income country. In addition, the model projected healthcare cost savings of US$ 42.8 million over the study period. These savings are likely to be particularly relevant in health systems where resources for cancer prevention and treatment remain constrained.

Comparison with data from other countries, including Thailand and settings in Europe and North America, suggests that the Kazakhstan estimates are plausible within the broader international context. For example, previous analyses from Thailand reported an ICER of approximately US$ 1400 per DALY averted for HPV vaccination [52]. The lower ICER values observed in the present study may reflect differences in model structure, epidemiological inputs, vaccine pricing, healthcare costs, and programme assumptions. Nevertheless, the findings suggest that HPV vaccination in Kazakhstan is likely to provide favourable value for money under the conditions modelled. These findings are also broadly consistent with results reported in lower- and middle-income countries with comparable healthcare settings.

Kazakhstan has previously experienced interruption of HPV vaccination because of parental refusal and limited public confidence. Under such conditions, vaccine availability alone may not be sufficient to ensure sustained population-level benefit. High uptake will also require effective communication strategies, continued public engagement, stable vaccine supply, maintenance of the cold chain, and long-term institutional support. International experience suggests that integration of vaccination with educational outreach and accessible delivery strategies can improve programme acceptance and coverage.

Several limitations should be considered when interpreting these findings. First, the model did not include indirect effects such as herd immunity or possible type replacement, which may have resulted in conservative estimates of programme impact. Second, the 10-year projection horizon may not fully capture the longer-term epidemiological and economic effects of HPV vaccination, particularly for cervical cancer outcomes that evolve over extended periods. Third, the analysis relied on adapted epidemiological inputs and on limited local screening and cost data, which may have influenced the precision of the estimates. Accordingly, the results should be interpreted as model-based projections rather than direct observations of programme performance.

In addition, recent WHO recommendations have introduced the option of a single-dose HPV vaccination schedule, which may offer advantages in terms of cost reduction and programme feasibility. The present analysis was based on a two-dose regimen to reflect the vaccination schedule currently implemented in Kazakhstan and to ensure consistency with national immunization practice. However, future modelling studies could evaluate the comparative cost-effectiveness of single-dose versus two-dose strategies under different coverage and pricing scenarios.

Despite these limitations, the study provides a useful framework for understanding the likely health and economic implications of HPV vaccination in Kazakhstan. Integration of HPV vaccination into the national immunization infrastructure, together with continued monitoring of vaccine uptake, programme delivery, and disease trends, will be important for evaluating real-world effectiveness over time. Further studies incorporating longer follow-up periods and more detailed local epidemiological and economic data would help refine these projections.

## 5. Conclusions

This study suggests that nationwide HPV vaccination of girls in Kazakhstan with the quadrivalent Gardasil-4 vaccine would likely be a cost-effective strategy associated with substantial reductions in HPV-related disease burden, mortality, and healthcare costs. The model supports the potential value of sustained HPV vaccination within the national prevention framework. Future work should focus on longer-term evaluation, incorporation of indirect effects, and generation of more detailed local data to strengthen evidence for programme monitoring and optimization.

## Figures and Tables

**Table 1:** Cost-effectiveness outcomes of HPV vaccination from governmental and societal perspectives.

Indicator	Government Perspective	Societal Perspective
Cost per DALY averted (US$)	533	1169
Vaccine programme costs (US$)	78,830,113	78,830,113
Healthcare costs averted (US$)	42,856,372	0
DALYs averted	67,445	67,445

**Table 2:** Reduction in healthcare costs and utilization associated with HPV vaccination from the governmental perspective.

Perspective	Metric	Without	With	Difference
Government	Total Healthcare Costs	62,797,375	19,941,003	42,856,372
	Visits	388,660	125,872	262,788
	Hospitalization-Related Costs (US$)	62,408,716	19,815,222	42,593,494

**Table 3:** Annual dynamics of key health and economic indicators under scenarios with and without HPV vaccination.

	Cases		Visits		Hosps.		Deaths		Vaccine Programme Costs	Healthcare Costs Averted	
Calendar year	Without	With	Without	With	Without	With	Without	With	Cost (m)	Govt	Society
2025	-	-	-	-	-	-	-	-	4.6	-	-
2026	-	-	-	-	-	-	-	-	9.1	-	-
2027	-	-	-	-	-	-	-	-	9.2	-	-
2028	-	-	-	-	-	-	-	-	9.3	-	-
2029	-	-	-	-	-	-	-	-	9.3	-	-
2030	9	3	2	1	0	0	0	0	9.3	0	-
2031	17	5	4	1	1	0	1	0	9.3	0	-
2032	26	8	6	2	1	0	1	0	9.3	0.01	-
2033	35	11	8	3	1	0	1	0	9.3	0.01	-
2034	44	14	10	3	2	1	2	1	9.3	0.01	-

Note: Values in the columns, “Cases”, “Visits”, “Hospitalizations”, and “Deaths”, represent annual model-predicted events under scenarios with and without HPV vaccination. Vaccine programme costs are presented in million USD (discounted values). Healthcare costs averted are shown from governmental and societal perspectives. Abbreviations: Hosps., hospitalizations; Govt, governmental perspective; Society, societal perspective.

## Data Availability

Data used in this study were obtained from publicly available sources, including WHO databases, GLOBOCAN 2020, and United Nations Population Division (UNPOP) datasets. The study results are based on model outputs generated using the UNIVAC tool with country-specific input parameters. All source data are publicly accessible through the cited databases, and the main model assumptions and inputs are described in the manuscript. Additional analytical details are available from the corresponding author upon reasonable request.

## Abbreviations

The following abbreviations are used in this manuscript:

CIN	Cervical Intraepithelial Neoplasia
CEAC	Cost-Effectiveness Acceptability Curve
DALY	Disability-Adjusted Life Year
GDP	Gross Domestic Product
HPV	Human Papillomavirus
ICER	Incremental Cost-Effectiveness Ratio
ICC	Invasive Cervical Cancer
ICEP	Incremental Cost-Effectiveness Plane
PSA	Probabilistic Sensitivity Analysis
UNIVAC	Universal Vaccination Impact and Cost-Effectiveness Assessment
USD	United States Dollar
WTP	Willingness-To-Pay
WHO	World Health Organization
WUENIC	WHO/UNICEF Estimates of National Immunization Coverage
